# Enhanced high-frequency absorption of anisotropic Fe_3_O_4_/graphene nanocomposites

**DOI:** 10.1038/srep25075

**Published:** 2016-05-04

**Authors:** Yichao Yin, Min Zeng, Jue Liu, Wukui Tang, Hangrong Dong, Ruozhou Xia, Ronghai Yu

**Affiliations:** 1 School of Materials Science and Engineering, Beihang University, Beijing 100191, China

## Abstract

Anisotropic Fe_3_O_4_ nanoparticle and a series of its graphene composites have been successfully prepared as high-frequency absorbers. The crystal structure, morphology and magnetic property of the samples were detailed characterized through X-ray diffractometer (XRD), transmission electron microscopy (TEM) and vibrating sample magnetometer (VSM). The high-frequency absorbing performance of the composites is evaluated within 2.0–18.0 GHz. Combining reduced graphene oxide (RGO) to Fe_3_O_4_ helps to adjust the permittivity and permeability of the composite, balance the dielectric loss and magnetic loss, consequently improve the absorbing performance in view of the impedance matching characteristic. The optimal reflection loss of the pure Fe_3_O_4_ sample reaches −38.1 dB with a thickness of 1.7 mm, and it increases to −65.1 dB for the sample grafted with 3 wt.% RGO. The addition of proper content of RGO both improves the reflection loss and expands the absorbing bandwidth. This work not only opens a new method and an idea for tuning the electromagnetic properties and enhancing the capacity of high-efficient absorbers, but also broadens the application of such kinds of lightweight absorbing materials frameworks.

Recently, high-frequency wave absorption materials have attracted a great deal of attention because of their potential applications in the fields of wireless data communication, mobile phones, radar systems, local area networks, satellite television and self-concealing^1^,^2^,^3^,^4^. Excellent absorption materials should have strong wave attenuation abilities as well as a wide absorption bandwidth^5^. The attenuation of the electromagnetic wave is mainly in the form of magnetic or dielectric loss by transforming it into thermal energy^6^. Thus, adjusting the electromagnetic parameters of the materials combining kinds of lossing principles and keeping a balance between the dielectric loss and magnetic loss in view of the impedance matching characteristic will improve the absorbing performance. Moreover, considering the bandwidth, it is important for an absorber to exhibit multiple resonance phenomena in the frequencies which may contribute to fulfil wide absorbing range.

Ferrite material has been widely used in magnetic sensor, magnetic resonance, electro-magneto-rheological fluid^7^, microwave absorption^8^, and so on. So many works about its absorbing properties have been conducted and it has been proved to be a prosperous family for wave absorption. For instance^9^, the minimum reflection loss value of a conductive PANI/MnFe_2_O_4_ nanocomposite is −15.3 dB at 10.4 GHz with the thickness of 1.4 mm. And it is −12.0 dB at 11.3 GHz with the thickness of 1.5 mm for another conductive PPy/MnFe_2_O_4_ nanocomposite scattering in resin acrylic^10^. For one more example, the optimal reflection loss value of the coin-like α-Fe_2_O_3_@CoFe_2_O_4_ core-shell composite can reach −60 dB at 16.5 GHz with a thickness of 2.0 mm^11^. As a wave absorption material, Fe_3_O_4_ have been studied extensively for its excellent absorption properties by virtue of strong permeability and relative high resistivity. Many Fe_3_O_4_ composites also have been reported in recent years: Fe_3_O_4_@ZnO sphere decorated graphene^12^, Fe_3_O_4_/TiO_2_ core-shell nanotubes^13^, Fe_3_O_4_@TiO_2_ yolk–shell microspheres^14^, fluorinated polybenzobisoxazole/silica-coated magnetic Fe_3_O_4_ nanocomposities^15^, Fe_3_O_4_/SiO_2_ nanorods^16^, graphene@Fe_3_O_4_ nanocluster@carbon@MnO_2_ nanosheet array composites^17^, superparamgnetic Fe_3_O_4_ nanocrystals^18^, Fe_3_O_4_@C core-shell nanotubes^19^, Fe_3_O_4_@metal–organic framework^20^, 3D Fe_3_O_4_ nanocrystals decorating carbon nanotubes^21^.

Graphene has been applied as a new wave absorption material because of its desirable physical and chemical properties. Nevertheless, pure graphene has very weak EM wave absorption properties^22^,^23^. Many researchers have synthesized magnetic nanoparticles coupled with graphene that can highly improve the absorption performance^24^,^25^. Wang and *co*-workers^26^ synthesized graphene/Fe_3_O_4_/SiO_2_/NiO hierarchical nanosheets, of which the minimum reflection loss was up to −51.5 dB at 14.6 GHz with a thickness of only 1.8 mm and the absorption bandwidth with a reflection loss below −10 dB ranged from 12.4–17.5 GHz. Zhu *et al.*^27^ reported a graphene-carbonyl iron cross-linked composite of 3.0 mm with a minimum reflection loss reaching −52.46 dB at 9.46 GHz.

The size, shape and composite structure play importance roles on the absorption properties of the ferrite materials^28^,^29^,^30^,^31^. To further investigate the wave absorption property of the Fe_3_O_4_ nanocomposites, we synthesized anisotropic α-Fe_2_O_3_ nanoparticles by a facile hydrothermal process. The particles shown spindle-like shape and were then combined with graphene oxide (GO) to form α-Fe_2_O_3_/GO nanocomposites by different ratio. The anisotropic α-Fe_2_O_3_ nanoparticles were homogeneously dispersed in the graphene aqueous suspension and embedded into the graphene network. Finally, the Fe_3_O_4_/RGO nanocomposites were obtained after annealing in H_2_/Ar (5%:95%) atmosphere for 2 hours at 500 °C.

## Experimental

### Synthesis of spindle-like α-Fe_2_O_3_ nanoparticles

The monodispersed spindle-like α-Fe_2_O_3_ nanoparticles was parallel to the literature through a refluxing process^32^,^33^. Briefly, 1.08 g FeCl_3_ · 6H_2_O, 10 mg Na_2_HPO_4_ · 12H_2_O and 200 mL deionized water were directly added into a round-bottomed flask. The mixture was heated to 110 °C and refluxed under continuous stirring for 24 hours. After cooling down to room temperature, a red brown homogeneous suspension containing α-Fe_2_O_3_ nanoparticles was achieved. The final samples were firstly centrifuged, and then washed with deionized water and ethanol three times, respectively.

### Synthesis of Fe_3_O_4_/RGO nanocomposites

**Figure 1:**
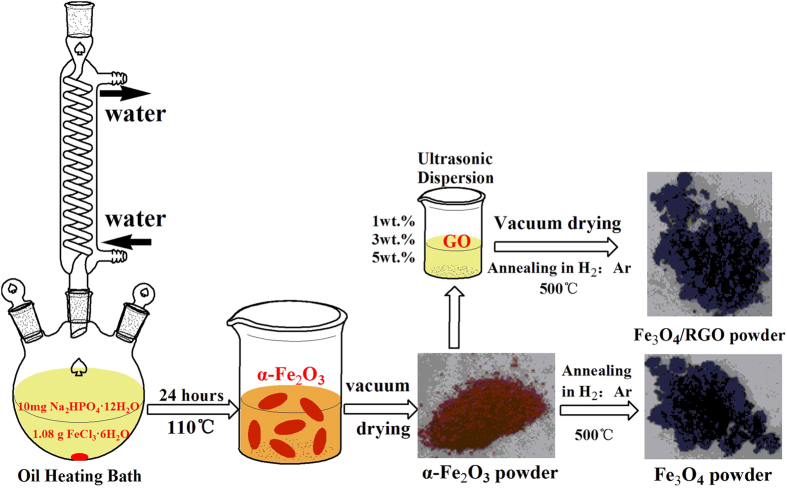
Illustration of the synthetic protocol for the Fe_3_O_4_/RGO nanocomposites.

A series of Fe_3_O_4_/RGO nanocomposites were performed by a simple ultrasonic-dispersion method, illustrated in Fig. 1. Typically, 3.0 mg GO powders were added to 50 ml of deionized water and sonicated for 1 hour. Then 0.3 g α-Fe_2_O_3_ nanoparticles were added into the above GO suspension and sonicated for another 1 hour. The precipitates were collected by centrifugation, followed by annealing in H_2_/Ar atmosphere (5%:95%) for 2 hours at 500 °C to obtain Fe_3_O_4_/RGO (1.0 wt.%) nanocomposites. The other two Fe_3_O_4_/RGO nanocomposites were achieved by modifying the contents of GO to 9.3 mg and 15.8 mg (3 wt. % and 5 wt. %), respectively.

### Characterization

The crystal structure of the samples was analyzed using X-ray diffractometer (XRD, Rigaku D/MAX-2500) with a Cu K_a_ irradiation (λ = 1.54178 Å, 40.0 kV, 150.0 mA), recorded from 5° to 90° (2θ) with a scanning step of 6°/min. Transmission electron microscopy (TEM, JEOL-2100) was used to observe the morphology, size and microstructure of the samples. Room-temperature magnetic properties of the samples were measured by a Riken vibrating sample magnetometer. The complex permittivity and permeability parameters of the composites were measured using an Agilent N5230C network analyzer in the range of 2.0–18.0 GHz, for which the samples containing 50 wt.% obtained composites and 50 wt.% wax were pressed into toroidal shapes (*Φ*_out_ = 7.00 mm and *Φ*_in_ = 3.04 mm).

## Results and Discussions

### Structure and morphology

**Figure 2:**
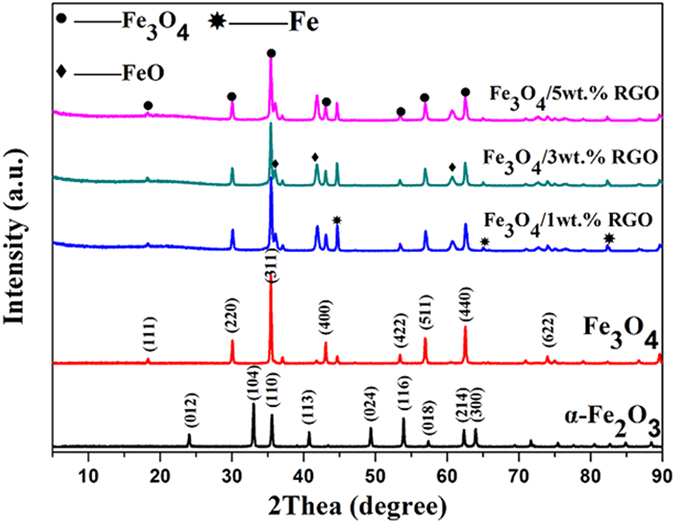
The XRD patterns of the samples obtained from different synthetic steps.

The crystalline structures of the as-prepared samples are presented by XRD patterns, shown in Fig. 2. Obviously, all XRD diffraction peaks belonging to crystalline α-Fe_2_O_3_ can be seen for the first step. Nine peaks at 24.1°, 33.1°, 35.6°, 40.8°, 49.4°, 53.9°, 57.4°, 62.3° and 63.9° are assigned to the reflections from the (012), (104), (110), (113), (024), (116), (018), (214) and (300) crystal planes (JCPDS card no. 24-0072), respectively, which is in good agreement with the reference data for α-Fe_2_O_3_ phase. No additional peaks belonging to other phases are observed, indicating the good crystallinity and high purity of the original α-Fe_2_O_3_ nanoparticles. After reduced for 2 hours in H_2_/Ar atmosphere, the diffraction peaks for the as-prepared particles are in good agreement with the data for the cubic spinel structured Fe_3_O_4_ (JCPDS card no. 65-3107), demonstrating this reduction method is efficient for the phase transformation from α-Fe_2_O_3_ to Fe_3_O_4_. After grafted on RGO, the intensity of diffraction peaks of the Fe_3_O_4_/RGO nanocomposites are weakened compared with that of the Fe_3_O_4_ due to the RGO cover. Compared with Figure S1, a weak and broad peak at around 20° is the typical pattern of amorphous carbon, indicating the RGO structures. Little diffraction peaks belonging to FeO at 36.0°, 41.8°, 60.7°, 72.7°, 76.6° (JCPDS card no. 06-0615) and Fe at 44.6°, 65.0°, 82.3° (JCPDS card no. 06-0696) can be detected in the composites, due to hydrogen as a reducing gas can more easily penetrate the gap within the multistage structures, which help further accomplish the phase transformations from Fe_3_O_4_ to some FeO and Fe.

**Figure 3:**
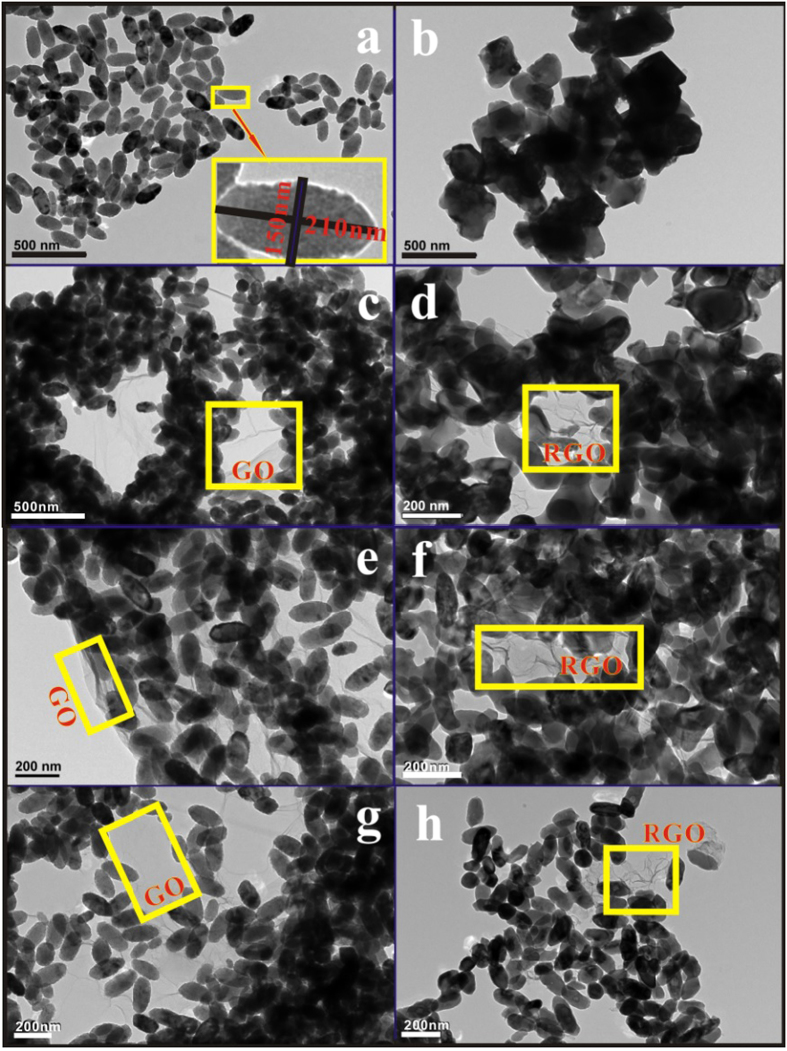
The TEM images of (**a**) the as-prepared α-Fe_2_O_3_, (**b**) Fe_3_O_4_ nanoparticles, (**c**) α-Fe_2_O_3_/1 wt.% GO, (**d**) Fe_3_O_4_/1 wt.% RGO, (**e**) α-Fe_2_O_3_/3 wt.% GO, (**f**) Fe_3_O_4_/3 wt.% RGO, (**g**) α-Fe_2_O_3_/5 wt.% GO and (**h**) Fe_3_O_4_/5 wt.% RGO nanocomposites.

**Figure 4:**
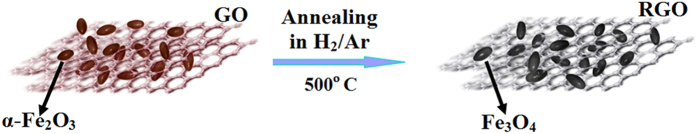
Skeleton of the synthesis process of the Fe_3_O_4_/RGO composite.

The TEM morphologies of a series of nanocomposites are shown in Fig. 3. The hydrolysis of the iron precursor with the help of Na_2_HPO_4_ leads to the monodispersed anisotropic spindle-like nanocrystalline α-Fe_2_O_3_ nanostructures, as shown in the inset of Fig. 3(a), with a average length of 200 nm and the outer diameter around 150 nm. After annealing treatment, the uniform dispersed spindle-shape particles are mainly destroyed and developed to bigger irregular Fe_3_O_4_ structures (Fig. 3(b)). Increasing the contents of GO, the α-Fe_2_O_3_ nanoparticles can be more evenly dispersed in the graphene layers. The α-Fe_2_O_3_ nanoparticles react with some polar functional groups such as hydroxyl, carboxyl or oleylamine and are slightly aggregated and grafted on the GO surfaces, ensuring the integrity of spindle-shaped Fe_3_O_4_ structures after annealing. From the TEM images, the GO has the typical crumpled structures with abundant wrinkles on the surface and scrolling on the edge of the nanosheets. Besides, the GO nanosheets are almost transparent in TEM pictures, indicating that they are very thin. The uniform spindle-like α-Fe_2_O_3_ nanoparticles shown in Fig. 3(c,e,g) are anchored onto the surfaces of graphene sheets, forming a cross-linked framework structure illustrated as Fig. 4. On one hand, the spindle-like nanoparticles prevent the GO sheets from folding; On the other hand, the curly GO sheets help to separate the α-Fe_2_O_3_ nanoparticles and consequently, prevent the Fe_3_O_4_ nanoparticles from agglomerating during annealing to form a homogeneous dispersion as shown in Fig. 3(b,d,h). When the amount of GO increases to 5 wt.%, the final Fe_3_O_4_ nanoparticles remain spindle-like morphologies as the original α-Fe_2_O_3_ nanoparticles, dispersing on the RGO nanosheet network.

### Magnetic properties

**Figure 5:**
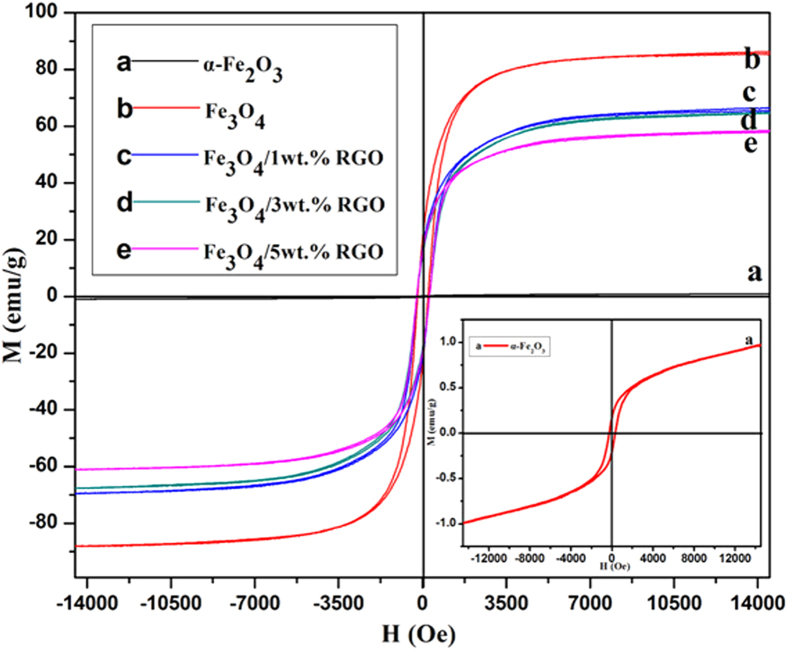
Room-temperature magnetic hysteresis loops of the samples.

The room-temperature magnetic hysteresis (*M-H*) loops in Fig. 5 show the magnetic variation for the samples from different processes. The value of magnetization saturation (*M*s) for the pure α-Fe_2_O_3_ nanoparticles is only 0.98 emu/g. It increases to 86.56 emu/g for the Fe_3_O_4_ then gradually deceases to 59.38 emu/g as increasing the ratio of the non-magnetic RGO nanosheets to 5 wt.%. Besides, the remnant magnetization (*M*r) and coercivity (*H*c) of the samples are also shown as Table 1, do not reveal much variation after combining with RGO.

### Absorption properties

**Figure 6:**
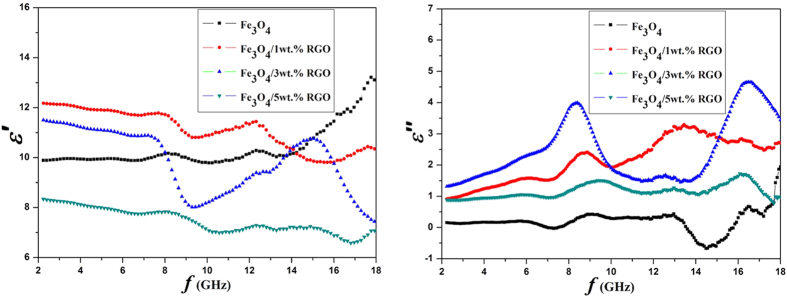
The frequency dependences of real and imaginary parts of the complex permittivities of the nanocomposites.

**Figure 7:**
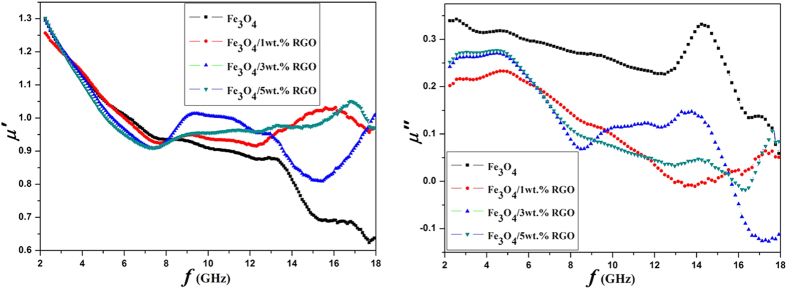
The frequency dependences of real and imaginary parts of the complex permeability of the nanocomposites.

A series of the pure Fe_3_O_4_ and Fe_3_O_4_/RGO nanocomposites are evaluated as high-frequency absorber. An efficient electromagnetic wave absorbing material should satisfy both strong absorbing and wide absorbing frequency band. The Fe_3_O_4_ units combined with RGO sheets to build a cross-linked framework may improve the wave absorbing performance. The frequency dependences of the complex permittivity (*ε*) and the complex permeability (*μ*) of the samples are shown in Figs 6 and 7, respectively. The real permittivity (*ε*′) and real permeability (*μ*′) represent the storage ability of electromagnetic energy, whereas the imaginary permittivity (*ε*″) and imaginary permeability (*μ*″) are connected with the energy dissipation or loss^34^,^35^.

Generally, the complex permittivity of the material shows frequency dispersion behaviour^28^,^36^. As shown in Fig. 6, the values of *ε*′ for Fe_3_O_4_ generally increase from 9.9 to 13.2 when the frequencies increase from 2.0 to 18.0 GHz, while the values of *ε*″ are almost under 1 and fluctuate as the frequencies increasing and reveal several resonance peaks. The behaviors should be attributed to the permittivity property and the special structure of the Fe_3_O_4_ nanoparticles. Since ε′ is an expression of the polarizability of a material, which consists of dipolar polarization and electric polarization at microwave frequency^17^. The high ε′ for Fe_3_O_4_ means high levers of the electric polarization and electric conductivity due to the electron transfer between Fe^3+^ and Fe^2+^ irons. And the resonance peaks in the *ε*″ curve demonstrates multi-relaxations also originating from the dipole polarization. After combined with RGO, the dielectric properties of the composites depend on that of each component and the interaction between. Particularly, the values of *ε*″ of a series of Fe_3_O_4_/RGO nanocomposites are higher than that of the pure Fe_3_O_4_. When the ratio of RGO is 3 wt.%, the *ε*″ curve have two high resonance peaks at 8.4 GHz and 16.5 GHz. The peaks root in the interfacial polarization, known as Maxwell–Wagner polarization in a heterogeneous media consisting of RGO and different conductivity or permittivity components^37^. The complex permittivity of the composite with 5 wt.% RGO possessing the lowest values compared with those of 1 wt.% and 3 wt.% RGO is owing to the isolated RGO sheets will connect to each other in the composite when the RGO content is high enough, leading to a reduction of the electric dipole as the similar phenomena reported before^27^.

Figure 7 shows the real part (*μ*′) and imaginary part (*μ*″) of the complex permeability of the Fe_3_O_4_ and Fe_3_O_4_/RGO composites. The *μ*′ of the composites generally decreases as the frequency increasing. It drops from 1.33 to 0.64 for the Fe_3_O_4_. For 1 wt.%, 3 wt.% and 5 wt.% RGO samples, it does from 1.27, 1.32, 1.33 to 0.97, 1.01, 0.97, respectively. Increasing the RGO ratio helps to remain the *μ*′ value at the frequencies above 8.0 GHz. The *μ*″ of the composites appear similar trend to the *μ*′ as the frequency increasing, showing large decrease in 4.7–8.5 GHz and serious fluctuations in the 8.5–18.0 GHz.

**Figure 8:**
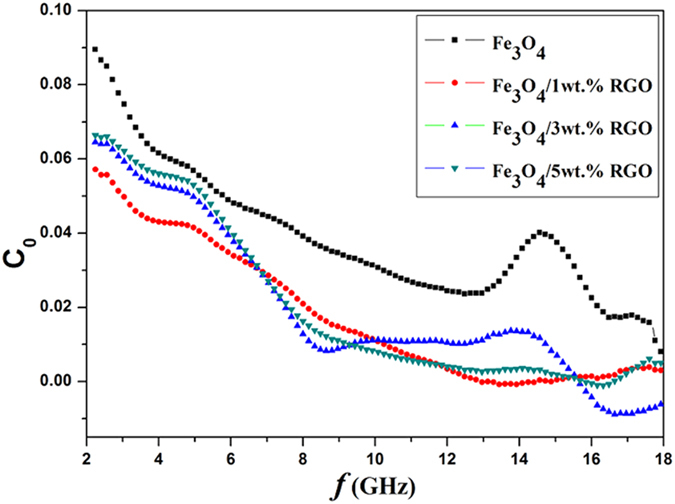
The frequency dependences of C_0_-f curves for the nanocomposites.

For most magnetic absorption materials, the magnetic loss could originate from the magnetic hysteresis, domain wall resonance, natural resonance, exchange resonance and eddy current effect^38^,^39^. The magnetic hysteresis loss is negligible in weak field. The domain wall resonance usually occurs at a much lower frequency range in multi-domain materials. The eddy current loss is another important factor for electromagnetic microwave absorption. It is related to the electric conductivity (*σ*) and thickness (*d*) of the samples, which can be expressed by *C*_0_^27^:


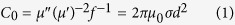


where *μ*_0_ is the permeability in a vacuum, σ is the electric conductivity of the material. If *C*_0_ is a constant with the change of frequency, we can say that the magnetic loss results from the eddy current loss^27^. As observed in Fig. 8, *C*_0_ decreases with the increasing frequency and have serious fluctuations in the whole frequency range, implying that the eddy current effect has no significant effect on the electromagnetic microwave absorption.

**Figure 9:**
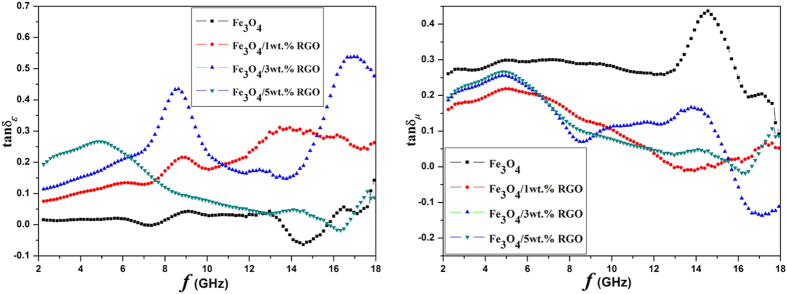
The dielectric loss and magnetic loss of the nanocomposites.

Dielectric loss and magnetic loss are the two mainly possible contributors for the absorption, which can be expressed as tanδ_*ε*_ = *ɛ*″/*ɛ*′ and tanδ_*μ*_ = *μ*″/*μ*′, respectively. It is very important to adjust the compatibility of the two kinds of loss to improve the absorption. Figure 9 shows the tanδ_*ε*_ and tanδ_*μ*_ of the samples. It is clear that the Fe_3_O_4_/RGO nanocomposites possess higher dielectric losses than that of the Fe_3_O_4_ sample. The enhanced dielectric loss could stem from the enhanced interfacial polarization between the Fe_3_O_4_ nanoparticles and RGO sheets. For magnetic loss, the values of the Fe_3_O_4_/RGO nanocomposites are lower than that of the Fe_3_O_4_, exhibiting the same variation trend as *μ*″. In view of the impedance matching characteristic for an absorber, well balance between the dielectric loss and magnetic loss could help to improve the absorbing performance, suggesting the lightweight graphene plays a key role in the improvement of the dielectric loss, which contributes to the absorption for the Fe_3_O_4_/RGO nanocomposites.

**Figure 10:**
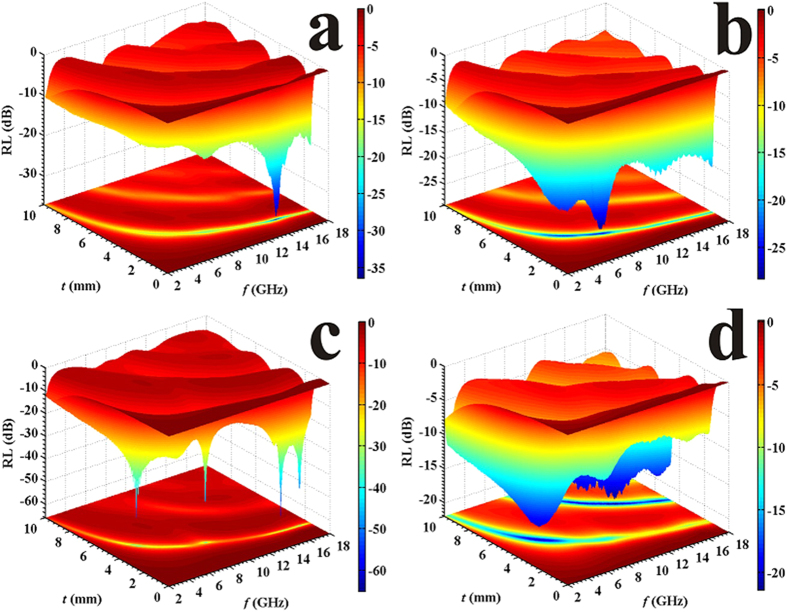
The reflection losses of the composites with different ratio between Fe_3_O_4_ and RGO.

The reflection loss (RL) values are calculated using the measured complex permittivity (*ε*_r_ = ε′ − *jε*″) and complex permeability (*μ*_r_ = *μ*′ − *jμ*″) at the given frequencies and the absorber thicknesses according to the transmission line theory as follows^40^:


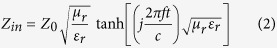



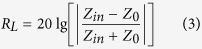



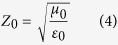


where *Z*_*o*_ (377 Ω) is the characteristic impedance of free space, *Z*_*in*_ is the input impedance of the absorber. *ε*_0_ and *μ*_0_ are the pemittivity and permeability of the free space, respectively. *f* is the frequency of the wave, *d* is the thickness of the absorber and *c* is the speed of light in free space. The results are illustrated in Fig. 10.

Figure 10 illustrates the reflection losses of the composites with different blending ratio. For the pure Fe_3_O_4_ composite, the image of reflection loss is shown in Fig. 10 (a). The peaks shift to high frequency with decrease of layer thickness and the optimal reflection loss can reach −38.1 dB at 14.8 GHz with a thickness of 1.7 mm. Combining Fe_3_O_4_ with 1 wt.% RGO, the optimal reflection loss is −28.2 dB at 8.5 GHz with a thickness of 2.7 mm (Fig. 10(b)). Adding 3 wt.% RGO makes it possess an optimal reflection loss of −65.1 dB at 15.2 GHz with a thickness of 1.7 mm (Fig. 10(c)). Further increasing the RGO to 5 wt.%, the optimal reflection loss becomes about −21.0 dB at 5.3 GHz with a thickness of 5.0 mm (Fig. 10(d)). Hence, addition of RGO with a proper content enhances the electromagnetic (EM) performance on the whole, which is ascribed to several aspects as indicated below. First, the RGO provides tremendous electric dipoles which react with high-frequency EM wave and convert EM energy to thermal energy. Second, the interfaces brought in have a dominant role in enhancing dielectric performance and also cause multiple reflections, further consuming the EM energy. Moreover, the introduction of RGO ameliorates the impedance matching to some degree so as to modify the EM absorbing performance^36^. In general, the composites with RGO exhibit multiple absorbing peaks at several points of the frequency and thickness. That is to say, RGO incorporated may expand the absorbing bandwidth and improve the reflection loss even with a smaller thickness.

**Figure 11:**
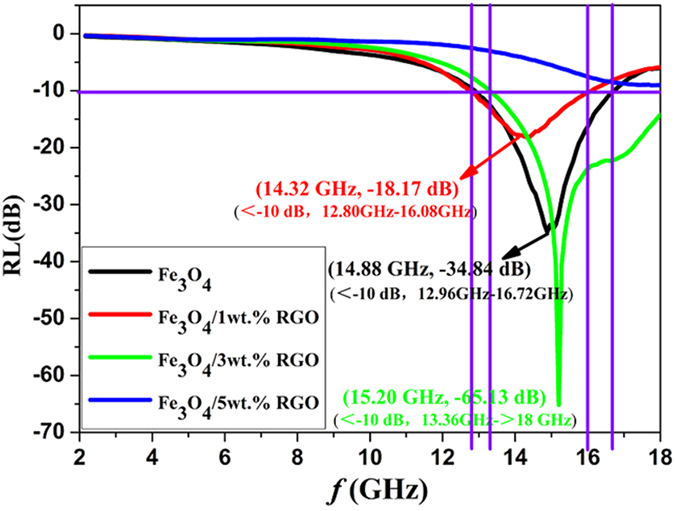
The bandwidths of the composites of 1.7 mm with different ratio between Fe_3_O_4_ and RGO.

When the thickness is 1.7 mm, the reflection losses versus frequency of the composites are shown in Fig. 11. The bandwidth of the Fe_3_O_4_/3 wt.% RGO composite for which the reflection loss is higher than −10 dB is from 13.4 GHz to over 18.0 GHz that larger than that of other samples, demonstrating wide range absorbing property. Therefore, adding proper content of RGO can increase both the reflection loss and the absorbing bandwidth, demonstrating the anisotropic Fe_3_O_4_/RGO nanocomposites are of high performance for high-frequency wave absorbing.

## Conclusions

In summary, anisotropic Fe_3_O_4_ nanoparticle and a series of Fe_3_O_4_/RGO nanocomposites have been successfully prepared. The Fe_3_O_4_/RGO nanocomposites exhibit high-performance microwave absorption properties over 2.0–18.0 GHz. Combining with RGO, the spindle-like Fe_3_O_4_ nanoparticles evenly dispersed in the graphene layers and are retarded from aggregating during annealing. The grafted composites possess higher dielectric losses than that of the pure Fe_3_O_4_ specimen, due to well balance between the dielectric loss and magnetic loss contribute to the high absorbing performance. The optimal reflection loss of the pure Fe_3_O_4_ composite is −38.1 dB at 14.8 GHz with a thickness of 1.7 mm. While it reaches −65.1 dB at 15.2 GHz with a thickness of 1.7 mm for the Fe_3_O_4_/3 wt.% RGO composite. The improved absorption arises from the synergy of dielectric loss and magnetic loss, as well as the enhancement of multiple interfaces among graphene. Adding proper content of RGO can increase both the reflection loss and the absorbing bandwidth, suggesting the Fe_3_O_4_/graphene nanocomposites are one kind of the prosperous candidates for EM wave absorbing.

## Additional Information

**How to cite this article**: Yin, Y. *et al.* Enhanced high-frequency absorption of anisotropic Fe_3_O_4_/graphene nanocomposites. *Sci. Rep.* **6**, 25075; doi: 10.1038/srep25075 (2016).

## Supplementary Material

Supplementary Information

## Figures and Tables

**Table 1:** Magnetic properties of the samples.

Sample	*M*s (emu/g)	*M*r (emu/g)	*H*c (Oe)
α-Fe_2_O_3_	0.98	0.18	285.11
Fe_3_O_4_	86.56	23.46	243.90
Fe_3_O_4_/1 wt.%RGO	67.46	19.94	287.19
Fe_3_O_4_/3 wt.%RGO	66.10	17.52	288.72
Fe_3_O_4_/5 wt.%RGO	59.38	18.62	290.99
