# Supplementary material for: Enhanced high-frequency absorption of anisotropic Fe3O4/graphene nanocomposites
Source: Sci Rep. 2016 May 4;6:25075. doi: 10.1038/srep25075 (PMC4855222; doi:10.1038/srep25075)
Supplement: Supplementary Information [file srep25075-s1.pdf]

# *Supplementary Information*

## Enhanced high-frequency absorption of anisotropic $Fe_3O_4$/graphene nanocomposites

Yichao Yin[1], Min Zeng*[1], Jue Liu[1], Wukui Tang[1], Hangrong Dong[1], Ruozhou Xia[1] & Ronghai Yu*[1]

[1]*School of Materials Science and Engineering, Beihang University, Beijing 100191, China.*

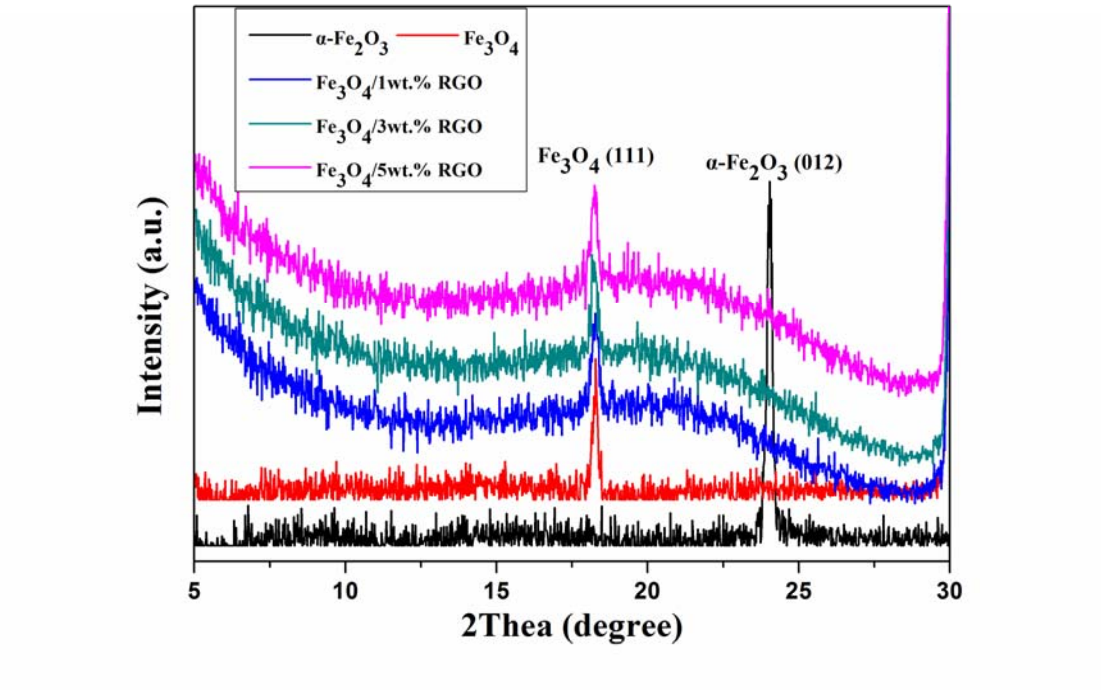


**Fig.S1** The XRD patterns of the samples obtained from different synthetic steps.
